# Supplementary material for: In the Drosophila germline H2Av and Arp6 suppress transposons by driving piRNA pathway expression
Source: bioRxiv. 2026 May 19:2026.05.19.726329. Preprint. [Version 1] doi: 10.64898/2026.05.19.726329 (PMC13228198; doi:10.64898/2026.05.19.726329)

bioRxiv preprint doi: https://doi.org/10.64898/2026.05.19.726329; this version posted May 21, 2026. The copyright holder for this preprint (which was not certified by peer review) is the author/funder, who has granted bioRxiv a license to display the preprint in perpetuity. It is made

Supplemental Figure Legends

Figure S1.
(A) RT-qPCR showing GLKD efficiency in His2Av-KD and Arp6-KD lines. Expression of His2Av and Arp6 are compared to the expression in the white-KD line. (B) Arp6 and H2Av GLKD causes slight reduction of male fertility. Respective hairpins were driven in the male germline by nos-Gal4-VP16 driver, males were crossed to wild-type females. (C) RT-qPCR analysis of select TEs indicates derepression of Het-A and Burdock upon H2Av and Arp6 GLKD. (Student's t-test, ****$p<0.0001$, **$p<0.01$, *$p<0.05$; error bars represent SD)

Figure S2.

(A) TEs derepressed upon H2Av or Arp6 GLKD do not show H2Av enrichment, except for the telomeric TEs. Scatterplot showing the relationship between levels of H2Av enrichment in control ovaries (log2fc input normalized counts at TSS) vs differential TE expression upon Arp6 and H2Av GLKD. (B) Pol II pSer5 accumulation at TE promoters exhibit modest increase in a subset of TEs upon H2Av and Arp6 GLKD. Pol II pSer5 ChIP-seq signal over TE promoters in control (shW) vs H2Av (left) and Arp6 (right) GLKD. Shown is input-normalized signal average of two biological replicates. Central dashed line shows x=y, outer dashed lines show 0.5 LFC ChIP/Input. In (A) & (B) significantly derepressed TE families (padj<0.05) are shown in black (nonsignificant in grey). Significance defined based on H2Av GLKD for both conditions. (C) H2Av's effect on TE silencing can be classified into piRNA-mediated and piRNA independent mechanisms. Shown are the 21 TEs with H2Av signal at their promoters, of which 15 show a decrease of a least 1 LFC in levels of targeting piRNA upon H2Av GLKD (top), while the other 6 do not. Fold change (log2FC shH2Av/shW) in TE transcript level based on RNA-seq (left), in piRNA level based on smallRNA-seq (middle) are indicated by differential expression color scale. Right side shows H2Av (in shW control) and Pol II pSer5 (in shW and shH2Av) ChIP-seq signal (scale showing log2 ChIP/input at the TSS +/- 1kb).

Figure S3.
(A) piRNA abundance at select clusters in White, Arp6 and His2Av GLKD. Total piRNAs (0 mismatches, uniquely mapping) mapping to each piRNA cluster, normalized

bioRxiv preprint doi: https://doi.org/10.64898/2026.05.19.726329; this version posted May 21, 2026. The copyright holder for this preprint (which was not certified by peer review) is the author/funder, who has granted bioRxiv a license to display the preprint in perpetuity. It is made available under a CC-BY 4.0 International license.

by miRNA read count for each sample. Statistical significance was determined by two-sided Student's t-test (*p<0.05, **p<0.01; n=2 biological replicates per condition). Error bars represent SD. (B) Quantification of differential cluster precursor expression from each 5kb window in the main piRNA-producing clusters (2 replicate DESeq2 differential analysis) in shH2Av (top) and shArp6 compared to shWhite, (C) Correlation between change in piRNA cluster precursor expression in shArp6/shW vs shH2Av/shW based on the RNA-seq quantifications in 5kb windows. (D) PolI II pSer5 ChIP-seq signal enrichment over input in the H2Av GLKD (left) and Arp6 GLKD (right) compared to the White GLKD control. Each datapoint represents aggregate signal over 5kb genomic tile that overlaps any cluster annotation, averaged for 2 replicates. (E) Average H2Av ChIP-seq signal enrichment over input in the shWhite control at 5kb genomic tiles overlapping clusters.

Figure S4.
(A) H2Av (left) and Arp6 (right) GLKD lead to global loss of Rhino. Input normalized Rhino ChIP signal in genome-wide 5kb tiles. Rhino loss in tiles overlapping piRNA clusters in purple compared to background tiles in grey (i.e., non-cluster and non-TE overlapping genomic tiles). Only tiles with H3K9me3 ChIP/input signal enrichment are shown (B) Levels of input-normalized Rhino and H3K9me3 ChIP signal (2 replicate average), in clusters and remaining non-cluster, non-TE genomic tiles (split into heterochromatin and euchromatin). (C) Input-normalized H3K9me3 and Rhino ChIP enrichment at select germline clusters. (D) Correlation of the change in Rhi signal at piRNA clusters upon Arp6 and H2Av GLKD when compared to shW. Each data point represents a 5kb tile that overlaps a cluster and has H3K9me3 ChIP/input signal enrichment in shW. (E) Cuff transcript is lost upon H2Av and Arp6 GLKD, while rhi mRNA is only reduced upon H2Av depletion. Confocal images of *in situ* hybridization chain reaction signal using *rhi* and *cuff* probes. (Scale bar - 20 μm).

Figure S5.
(A) Gene differential expression in shArp6 and shH2Av compared to shW is correlated. Significantly differentially expressed genes are shown in dark grey, with piRNA pathway genes highlighted in blue and labeled. (B) RT-qPCR showing loss of *gasz* and *mael* transcript in H2Av and Arp6 GLKD (Student's t-test, ***p<0.001, ****p<0.0001, ns – not significant, error bars represent SD). (C) Differential expression (log2FC KD/shW) of select somatic (black dots) and germline (orange x) factors upon Arp6 and H2Av GLKD compared to the control. (D) Pol II Chip-qPCR of selected piRNA pathway genes and RP49, as control (Student's t-test, *p<0.05, **p<0.01, ***p<0.001, ****p<0.0001, ns – not significant, error bars: SD). Primers were designed approx. 200bp downstream of TSS. (E) Representative single-replicate genome browser view (in IGV software) for AGO3 and Rhino showing RNA-seq expression levels in blue, H2Av ChIP-seq in red, and Pol II ChIP-seq in orange. Input signal for H2Av and Pol II ChIP are overlayed in grey. (F) Change in gene expression upon Apr6 GLKD does not correlate with H2Av loss at the TSS. Change in H2Av signal at gene TSS regions in the shArp6 knockdown plotted against the change in gene expression in shArp6. piRNA pathway genes are highlighted in blue.

Figure S6.
(A) H2Av and Pol II pSer5 accumulation correlate at TSSs of protein coding genes. Heatmap showing input normalized H2Av and Pol II pSer5 occupancy across all protein coding genes, averaged for 2 biological replicates. (B) Quantification of

bioRxiv preprint doi: https://doi.org/10.64898/2026.05.19.726329; this version posted May 21, 2026. The copyright holder for this preprint (which was not certified by peer review) is the author/funder, who has granted bioRxiv a license to display the preprint in perpetuity. It is made available under a CC-BY 4.0 International license.

frequent co-occurrence of Pol II pSer5 and H2Av ChIP signal (input normalized) surrounding the TSS of protein coding genes in the shW control. Percents displayed reflect the quantity of genes in each quadrant. Color scale depicts gene expression in shWhite (log10 CPM; 2 replicate average). (C) Heatmaps of H2Av ChIP signal distribution at genes that are upregulated, downregulated or unchanged upon H2Av GLKD. Color scale depicts log2(IP/input). (D) Metaplot of the H2Av ChIP-seq data displayed in (C). (E) H2Av enrichment at the 1kb region flanking the TSS at all expressed genes, categorized based on differential expression upon H2Av GLKD.

Supplemental Table Legends

Table S1. Primers

bioRxiv preprint doi: https://doi.org/10.64898/2026.05.19.726329; this version posted May 21, 2026. The copyright holder for this preprint (which was not certified by peer review) is the author/funder, who has granted bioRxiv a license to display the preprint in perpetuity. It is made available under a CC-BY 4.0 International license.

Andrasi et al. Figure S1

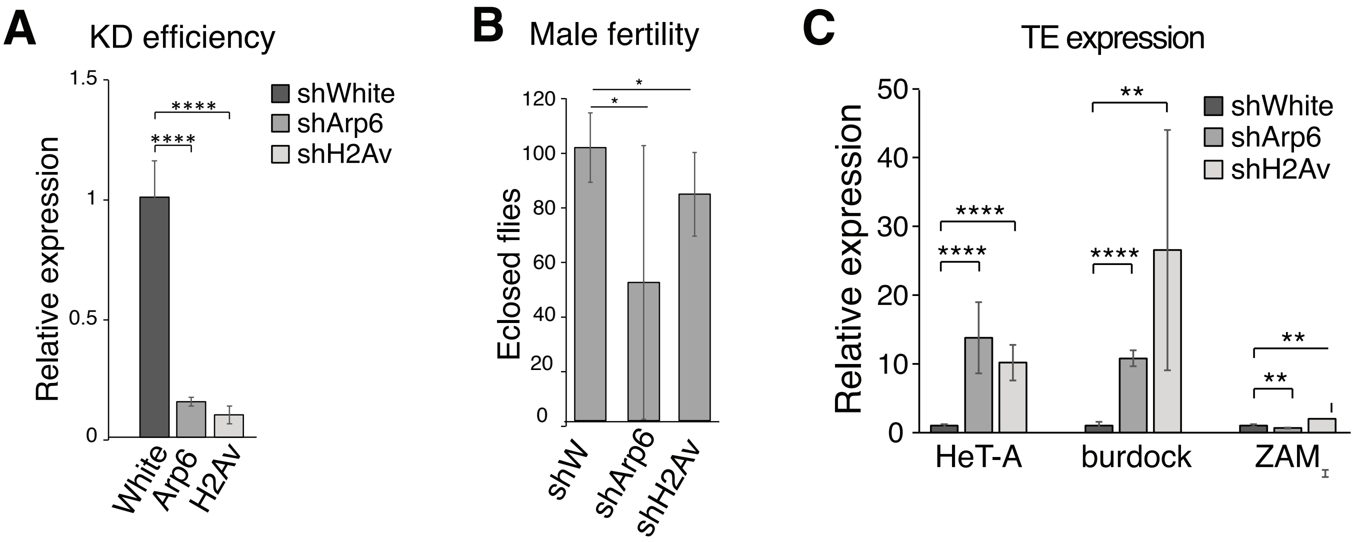

bioRxiv preprint doi: https://doi.org/10.64898/2026.05.19.726329; this version posted May 21, 2026. The copyright holder for this preprint (which was not certified by peer review) is the author/funder, who has granted bioRxiv a license to display the preprint in perpetuity. It is made available under a CC-BY 4.0 International license.

Andrasi et al. Figure S2

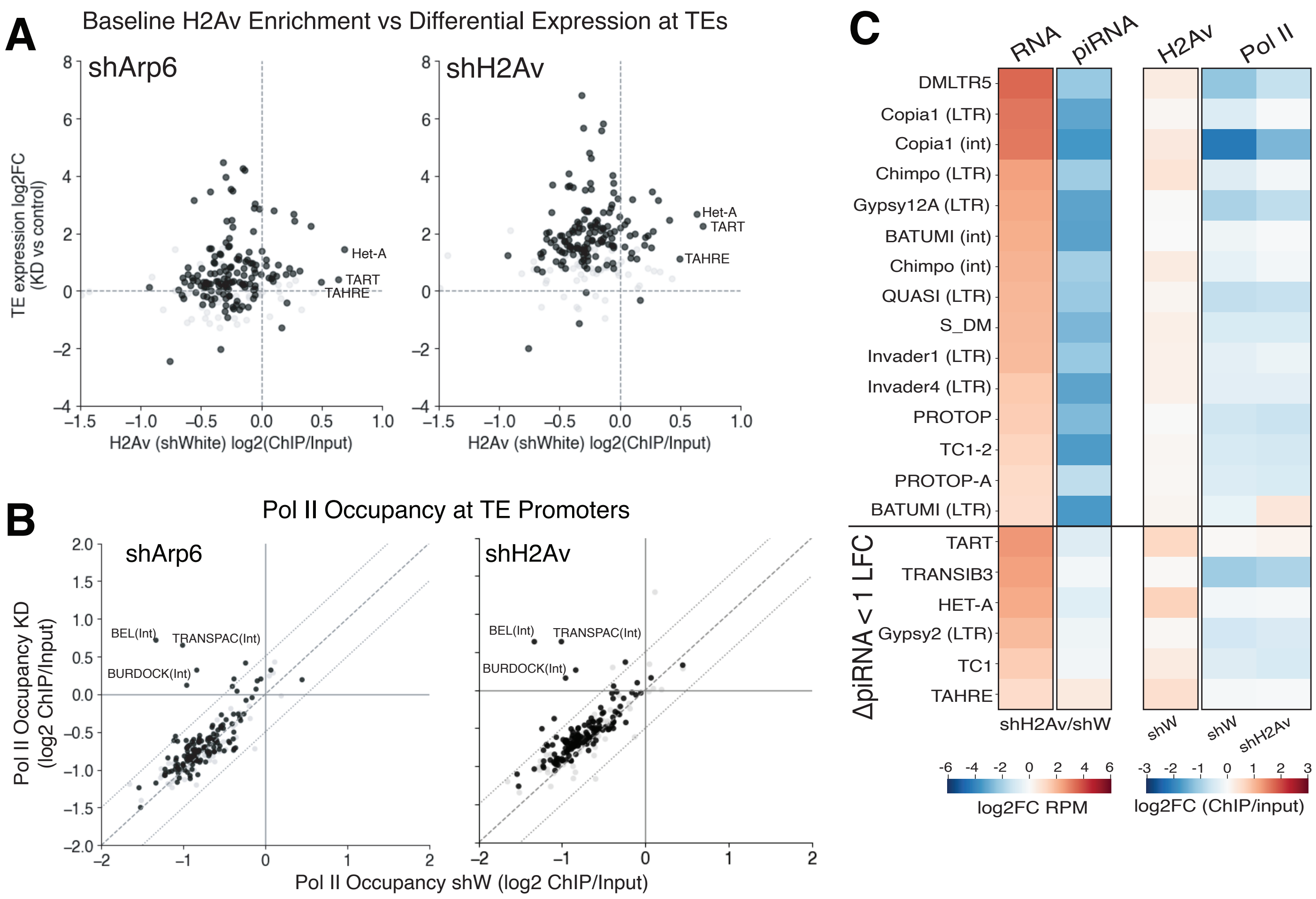

bioRxiv preprint doi: https://doi.org/10.64898/2026.05.19.726329; this version posted May 21, 2026. The copyright holder for this preprint
(which was not certified by peer review) is the author/funder, who has granted bioRxiv a license to display the preprint in perpetuity. It is made
available under a CC-BY 4.0 International license.

Andrasi et al. Figure S3

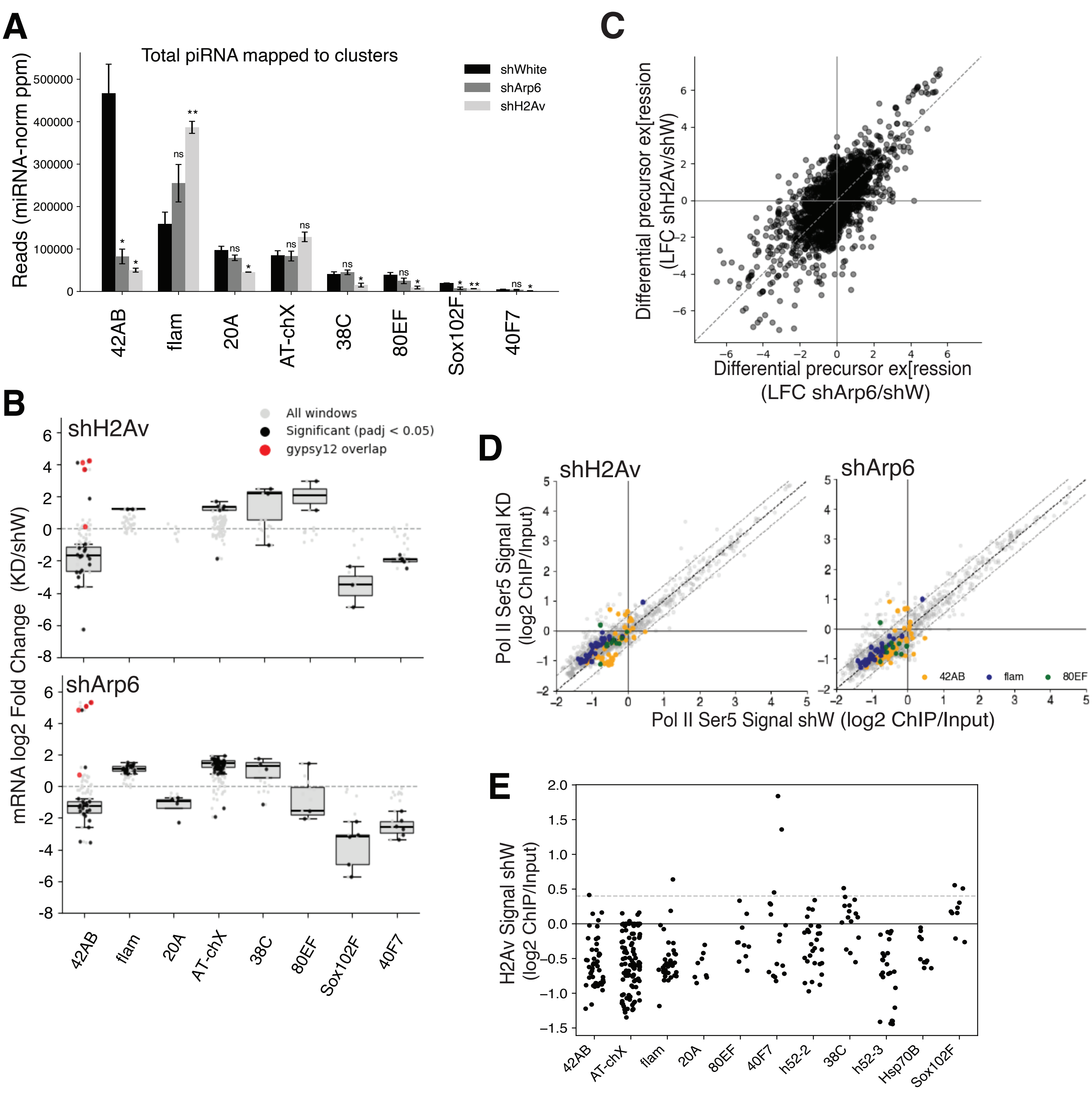

bioRxiv preprint doi: https://doi.org/10.64898/2026.05.19.726329; this version posted May 21, 2026. The copyright holder for this preprint (which was not certified by peer review) is the author/funder, who has granted bioRxiv a license to display the preprint in perpetuity. It is made available under a CC-BY 4.0 International license.

Andrasi et al. Suppl. Figure 4

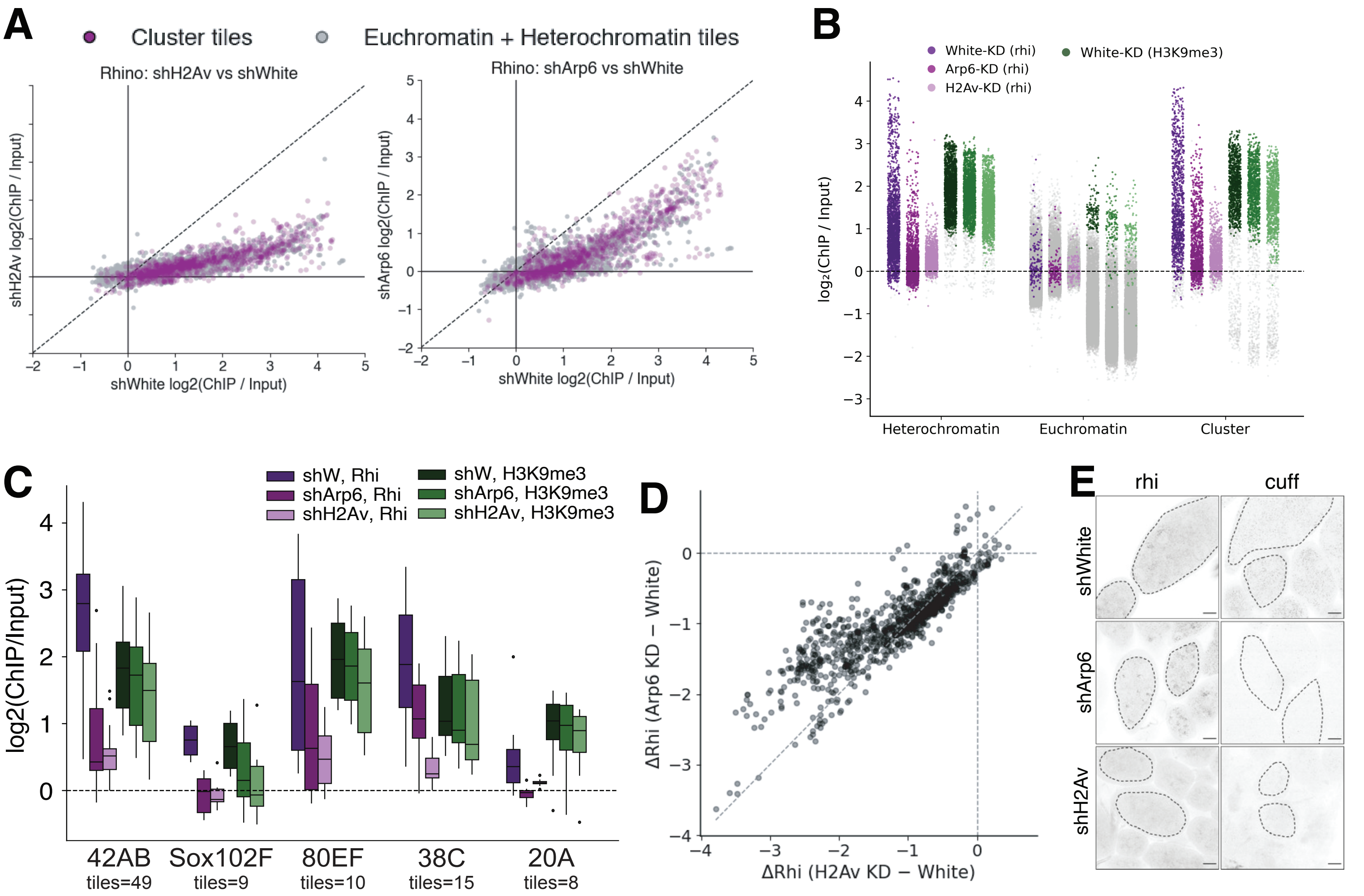

bioRxiv preprint doi: https://doi.org/10.64898/2026.05.19.726329; this version posted May 21, 2026. The copyright holder for this preprint (which was not certified by peer review) is the author/funder, who has granted bioRxiv a license to display the preprint in perpetuity. It is made available under a CC-BY 4.0 International license.

Andrasi et al. Suppl. Figure 5

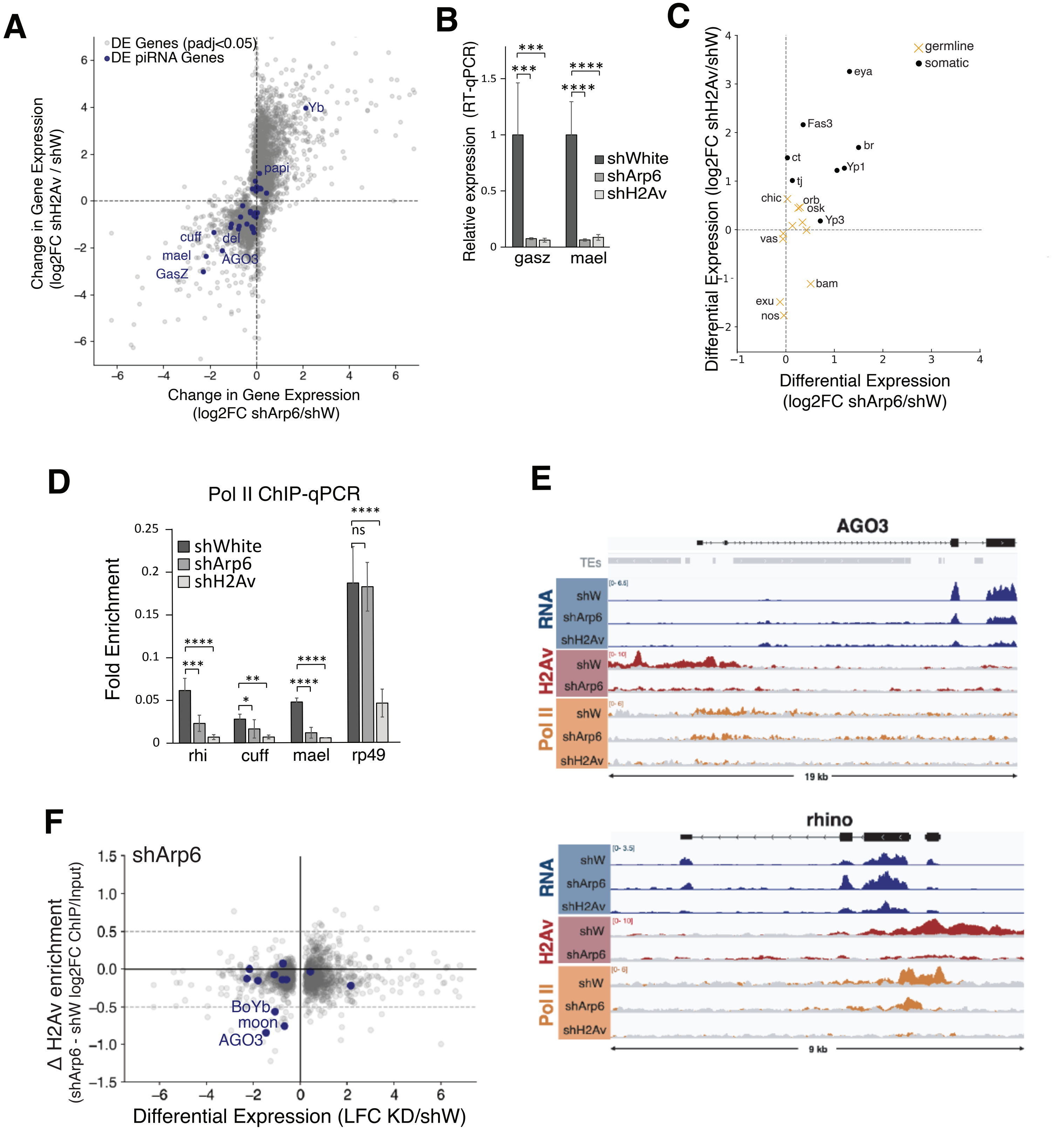

bioRxiv preprint doi: https://doi.org/10.64898/2026.05.19.726329; this version posted May 21, 2026. The copyright holder for this preprint (which was not certified by peer review) is the author/funder, who has granted bioRxiv a license to display the preprint in perpetuity. It is made available under a CC-BY 4.0 International license.

Andrasi et al. Suppl. Figure 6

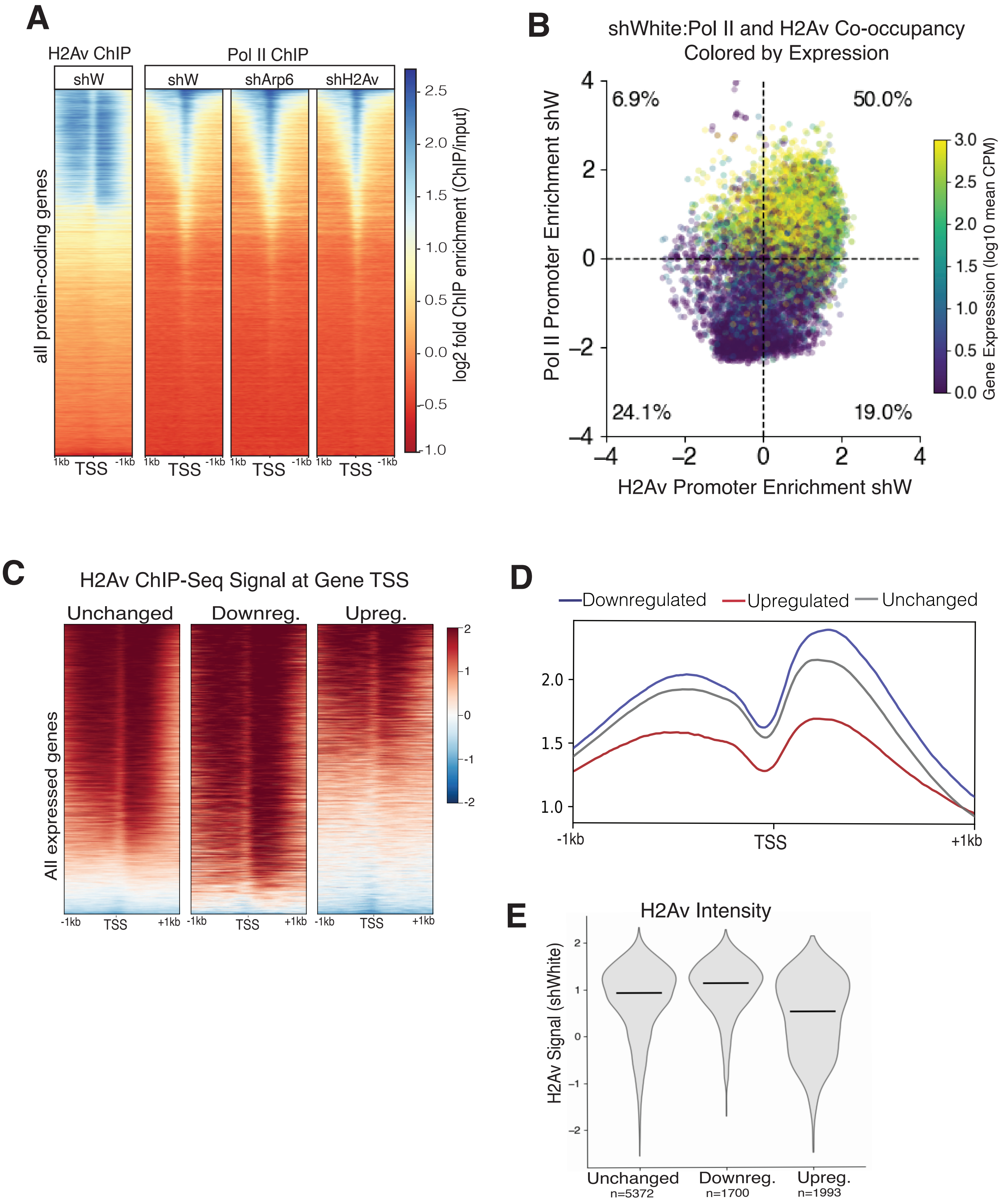

Supplement: Supplement 1 [file NIHPP2026.05.19.726329v1-supplement-1.pdf]
